# Sarcopenia using pectoralis muscle area and lymphocyte-to-monocyte ratio (LMR) are independent prognostic factors in patients for nonmetastatic breast cancer

**DOI:** 10.1097/MD.0000000000032229

**Published:** 2022-12-09

**Authors:** Haa-Na Song, Ju Yeon Kim, Jae Myung Kim, Ki Mun Kang, Hoon Sik Choi, Jin Hee Jeong, In Bong Ha, Bae-Kwon Jeong

**Affiliations:** a Division of Hematology-Oncology, Department of Internal Medicine, Gyeongsang National University of Medicine and Gyeongsang National University Hospital, Jinju, Korea; b Institute of Health Science, Gyeongsang National University, Jinju, Korea; c Department of Surgery, Gyeongsang National University of Medicine and Gyeongsang National University Hospital, Jinju, Korea; d Department of Radiation Oncology, Gyeongsang National University Changwon Hospital, Gyeongsang National University of Medicine, Changwon, Korea; e Department of Emergency Medicine, Gyeongsang National University School of Medicine and Gyeongsang National University Hospital, Jinju, Korea; f Department of Radiation Oncology, Gyeongsang National University of Medicine and Gyeongsang National University Hospital, Jinju, Korea.

**Keywords:** breast neoplasms, disease-free survival, inflammation, prognosis, sarcopenia

## Abstract

Sarcopenia is defined as loss of skeletal muscle mass and strength. This can lead to adverse clinical outcomes in patients with advanced cancer. The lymphocyte-to-monocyte ratio (LMR), a converted inflammatory response, is associated with poor prognosis in patients with malignancies. Herein, we examined the prognostic influence of sarcopenia status assessed by pectoralis muscle area (PMA), inflammatory status calculated by LMR, and its association with disease-free survival (DFS) in a cohort of women diagnosed with nonmetastatic breast cancer. A total of 293 patients with nonmetastatic breast cancer who underwent primary mass resection and radiotherapy between January 2011 and December 2017 were enrolled. The cross-sectional area of the muscle (cm^2^) at PMA was measured using computed tomography before radiation therapy. Baseline monocyte and lymphocyte counts were obtained from the complete blood count to calculate the LMR. Most of the patients (248/293, 84.6%) underwent breast conservation surgery. Lymph node involvement at diagnosis (hazard ratio [HR], 5.08; *P* < .001), low LMR (HR, 2.79; *P* = .007), and low PMA (HR, 3.80; *P* < .001) were independent poor prognostic factors in multivariate analysis. The mean DFS of sarcopenic and nonsarcopenic patients was 89.8 months and 118.8 months, respectively (*P < *.001). Sarcopenic patients with low LMR showed the worst outcomes, whereas nonsarcopenic patients with high LMR showed the best outcomes. Low PMA and low LMR were independent poor prognostic factors for DFS in patients with nonmetastatic breast cancer.

## 1. Introduction

Breast cancer is the most prevalent type of cancer among women worldwide^[1]^ and is the main cause of cancer-related mortality. Nevertheless, the overall survival rate of women with breast cancer is relatively high.^[2]^ Therefore, predicting the prognosis of patients with breast cancer is important for determining appropriate treatment. The tumor node metastasis staging system and its molecular subtypes are powerful tools for predicting prognosis.^[3,4]^ In addition, it has been reported that host factors, including age, race, menopausal status, and smoking history, can influence the prognosis of breast cancer.^[5–7]^

Sarcopenia is characterized by muscle failure, including skeletal muscle loss, which leads to reduced muscle function.^[8,9]^ Poor performance can lead to adverse clinical outcomes such as longer hospital stays and increased mortality. Sarcopenia can arise from old age and chronic inflammation caused by comorbidities, especially cancer.^[10]^

Substantial evidence indicates that chronic low-grade inflammation may be an important etiology of sarcopenia.^[11]^ Previous studies have shown that sarcopenia is associated with high levels of proinflammatory cytokines, which may be related to systemic inflammation that causes skeletal muscle degradation. This subsequently leads to decreased quality of life and shortened survival.^[12]^ The lymphocyte-to-monocyte ratio (LMR), a converted inflammatory response-related biomarker, is associated with worse prognostic significance in patients with various types of malignancies.^[13,14]^

To assess muscle mass, different imaging techniques can be used, such as magnetic resonance imaging,^[15]^ dual energy X-ray absorptiometry,^[16]^ and CT.^[17]^ CT is considered to be the standard imaging technique for assessing muscle mass and attenuation. It is assumed that the muscular cross-sectional area of the third lumbar vertebra (L3) is strongly correlated with total body muscle mass.^[18]^ However, abdominal imaging is not routinely performed in breast cancer patients. To easily assess sarcopenia in patients with nonmetastatic breast cancer, we measured the sum of peri-PMA.

Among patients with nonmetastatic breast cancer after resection of the primary breast cancer, chest CT scans are routinely performed for radiotherapy. Using CT imaging, the skeletal muscle area was measured as the sum of the surrounding pectoralis muscle area (PMA). To the best of our knowledge, no previous study has focused on the association between sarcopenia evaluated using PMA and the survival of patients with breast cancer. In addition, blood samples were routinely obtained from the patients, and the inflammatory status could be easily evaluated by calculating the individual LMR. Herein, we evaluated the prognostic influence of sarcopenia assessed by PMA and LMR, reflecting inflammatory status, on survival among patients with nonmetastatic breast cancer.

## 2. Methods

### 2.1. Patients

A total of 293 patients with nonmetastatic breast cancer, who underwent complete resection of the primary mass and postoperative radiotherapy between January 2011 and December 2017, were enrolled. The inclusion criteria were as follows: age ≥ 18 years, pathologically confirmed stage I–III breast cancer, and resection of the primary mass and/or chemotherapy and radiation therapy at Gyeongsang National University Hospital (GNUH).

### 2.2. Muscle mass measurement

We used simulated CT scans acquired for the initial radiation therapy planning to measure the skeletal muscle area. Two centrally trained radiation oncologists quantified the cross-sectional area of the muscle based on anatomical features of the PMA (cm^2^). PMA includes the sum of the pectoralis major and pectoralis minor muscles at the level of the fourth thoracic vertebra (T4).

The pectoral muscle volume of the chest wall, as opposed to that in primary breast cancer, was delineated from 2 consecutive axial images at the T4 level and divided by the CT slice thickness (3.75 mm) to calculate the skeletal muscle surface area. Varian Eclipse contouring software (Varian Medial Systems, Palo Alto, CA) was used to delineate and calculate the surface area of the pectoralis muscle according to the thresholds of Hounsfield units (−29 to + 150) of skeletal muscle tissue (Supplemental Figure 1, Supplemental Digital Content, http://links.lww.com/MD/I86).

### 2.3. Clinical data collection and definitions

Data were collected and analyzed via a retrospective chart review. Patients without primary pathological immunohistochemical information or primary clinical patient data were excluded. Breast cancer stage at diagnosis; pathologic findings such as immunohistochemical staining for estrogen receptor, progesterone receptor, and human epidermal growth factor receptor 2 (HER2); and breast cancer treatment data were obtained from the participants’ medical records. Baseline monocyte and lymphocyte counts were obtained from the complete blood count to calculate the LMR. Clinical data of the enrolled patients were also collected, including height, weight, body mass index, body surface area, and comorbidities, such as diabetes and dyslipidemia.

### 2.4. Definition of sarcopenia and systemic inflammation

**Figure 1.**
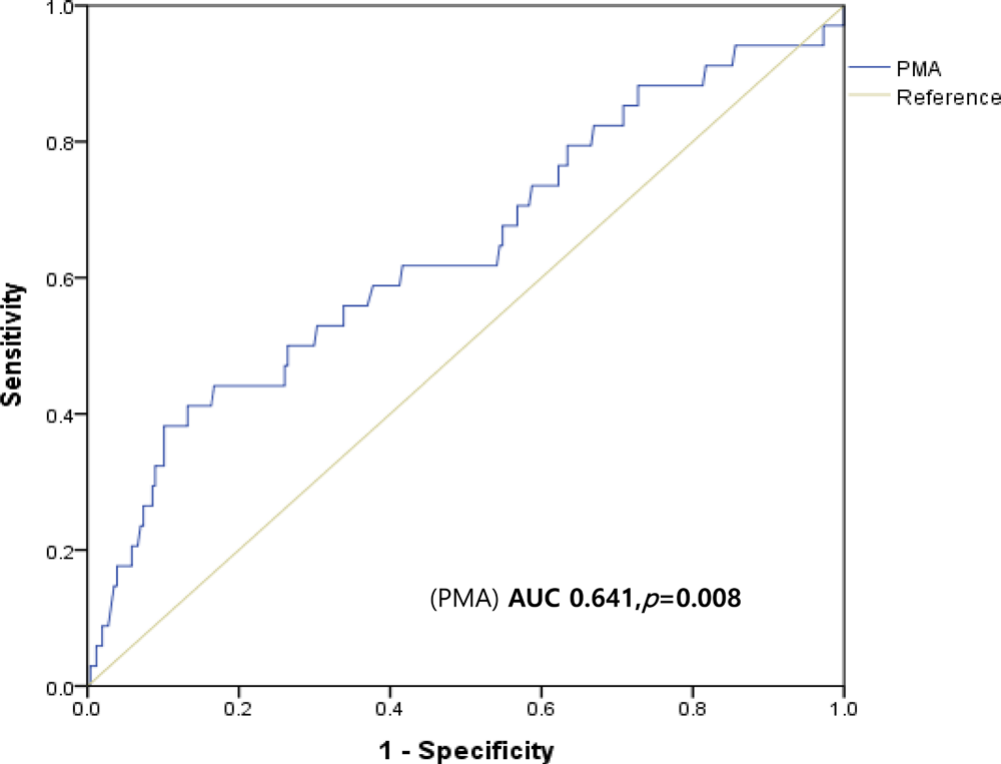
Receiver operating characteristic (ROC) curve by pectoralis muscle area (PMA). The cut-off value for sarcopenia was calculated using the ROC curve of PMA.

To assess the discriminatory ability of sarcopenia for prognostic prediction, a receiver operating characteristic (ROC) curve was constructed within the studied patient population and the area under the curve was calculated. The optimal cutoff value for the corresponding values was determined from the curve for the given sensitivity and specificity. Sarcopenia was defined as a PMA < 19.25 cm^2^. The cutoff value for sarcopenia was calculated using the ROC curve (Fig. 1). Furthermore, systemic inflammatory conditions were defined as an LMR < 3.95 based on the ROC curve.

### 2.5. Statistical analysis

Standard descriptive and analytical methods were used to define the demographic and baseline clinical characteristics of patients. Disease-free survival (DFS) was defined as the time from the date of diagnosis to the date of documented disease recurrence. Overall survival (OS) was defined as the time from diagnosis to the date of death. Kaplan–Meier estimates were used in the analysis of time-to-event variables, and 95% confidence intervals (CIs) for the median time-to-event were computed. Survival comparisons using univariate analysis were performed using the log-rank test. Cox’s proportional hazards model was used for multivariate analyses. Statistical significance was set at a *P*-value of less than .05, and all *p*-values corresponded to 2-sided tests. Statistical data were obtained using the SPSS software version 22 (SPSS Inc., Chicago, IL).

### 2.6. Ethics approval

The study protocol was approved by the institutional review board of GNUH (GNUH 2021-08-024). Because this study was a retrospective database review without any intervention or use of human specimens, the requirement for informed consent was waived.

## 3. Results

### 3.1. Patient characteristics

The baseline demographic features of the 293 patients are summarized in Table 1. All eligible patients were female, and the median age of the patients was 51 years (range, 26–81 years). Based on tumor node metastasis staging, approximately half of all patients were diagnosed as T1 or N0 (164/293, 56% and 169/293, 57.7%, respectively). Two-thirds of the patients (197/293, 67.2%) were diagnosed with stage IIA or lower disease based on the American Joint Committee on Cancer (AJCC) 8^th^ staging system. Most patients (248/293, 84.6%) underwent breast conservation surgery. The median PMA of the patients was 16.05 cm^2^ (range, 7.2–29.08 cm^2^). There was no significant association between low PMA and low LMR (Table 1).

**Table 1:** Baseline clinical features of 293 enrolled patients.

Characteristics	No. of patients	(%)
Age, year, median (range)	51 (26–81)	
Body mass index, median (BMI, kg/m^2^, range)	23.8 (17.6–38.1)	
Obesity based on BMI ≥ 23		
Yes	166	56.6
No	127	43.4
DM		
Yes	30	10.2
No	263	89.8
Sarcopenia based on PMA		
Yes	37	12.6
No	256	87.4
Systemic inflammation based on LMR		
Yes	74	25.3
No	219	74.7
T stage		
T1	164	56
T2	118	40.3
T3	9	3.0
T4	2	0.7
N stage		
N0	169	57.7
N1	65	22.2
N2	33	11.3
N3	26	8.8
Stage (AJCC 8^th^ edition)		
IA	17	5.8
IB	31	10.6
IC	71	24.2
IIA	78	26.6
IIB	32	10.9
IIIA	37	12.6
IIIB	1	0.4
IIIC	26	8.9
Operation methods		
Modified radical mastectomy (MRM)	45	15.4
Breast conserving surgery (BCS)	248	84.6
Chemotherapy		
Yes	245	83.6
No	48	16.4
Luminal type		
Luminal A	174	59.4
Luminal B	37	12.6
HER2-enriched	26	8.9
Basal-like	56	19.1
Duration of follow-up, month, median (range)	60.1 (23.7–122.1)	

AJCC = American Joint Committee on Cancer; HER2, human epidermal growth factor receptor 2, LMR = lymphocyte-to-monocyte ratio, PMA = pectoralis muscle area.

### 3.2. Survival analysis

Over a median follow-up duration of 60.1 (23.7–122.1) months, 34 patients relapsed and 17 patients died. For all 293 patients, the mean DFS and OS were 109.7 (95% CI, 105.8–113.6) and 115.6 (95% CI, 112.7–118.6) months, respectively. Median DFS and OS were not reached during the follow-up period.

**Figure 2.**
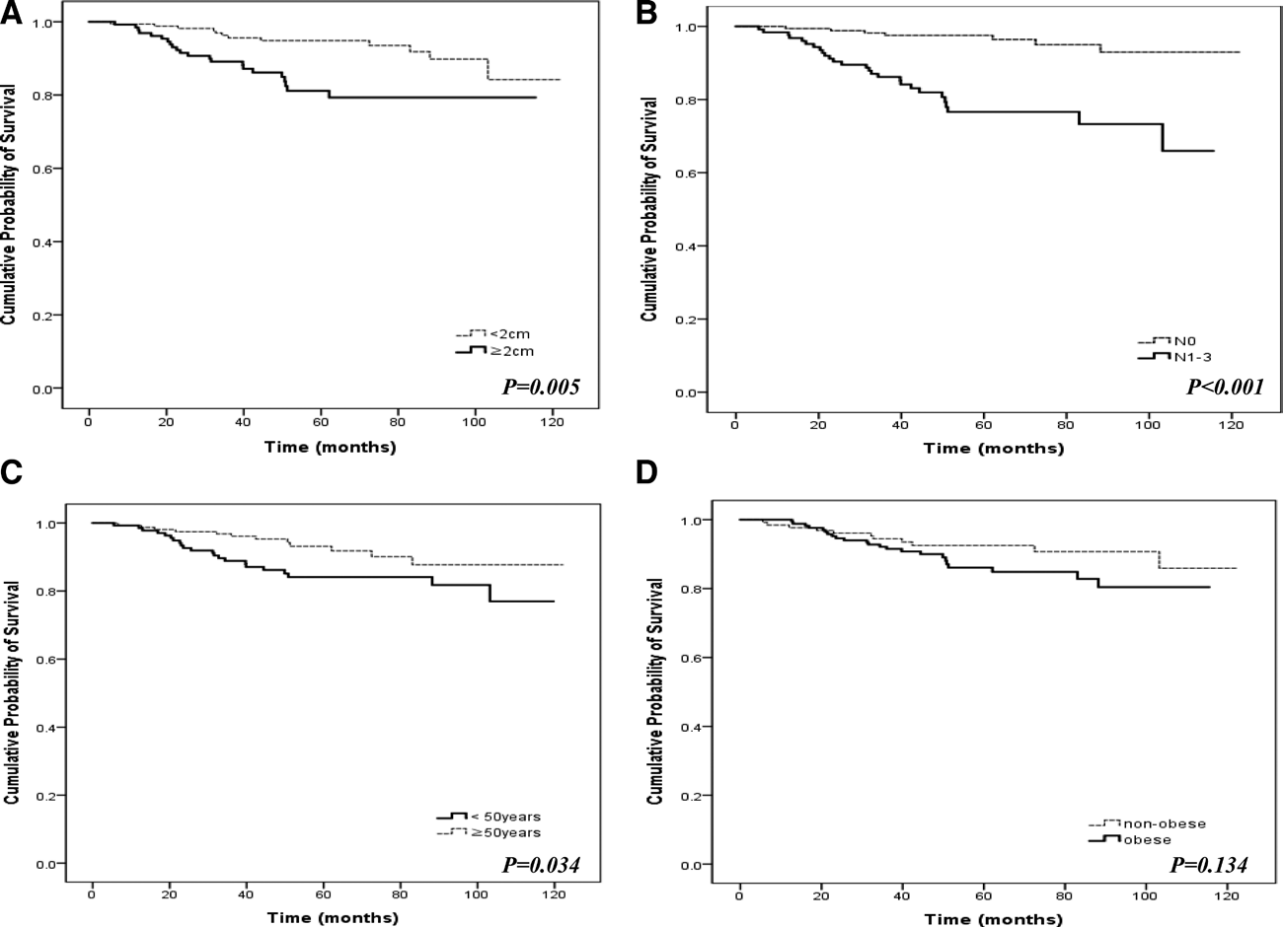
Kaplan–Meier curves for disease-free survival (DFS) by (A) tumor size, (B) lymph node status, (C) age, and (D) obesity.

We evaluated DFS according to the tumor characteristics and patient factors. Patients with tumor size > 2 cm and those who had cancer cells in their lymph nodes had significantly shorter DFS (mean 98.6 vs 114.2 months, *p = *-0.005 and 93.8 vs 117.6 months, *P < *.001, respectively; Figure 2a and 2b). Patients aged < 50 years had significantly shorter DFS; however, there was no significant difference in DFS according to obesity (103.7 vs 113.4 months, *p = *−0.034, and 102 vs 112.9 months, *p = *−0.134, respectively; Fig. 2c and 2d).

We evaluated the prognostic effect of systemic inflammatory factors, including C-reactive protein level, neutrophil/lymphocyte ratio, and platelet/lymphocyte ratio. However, there was no statistically significant relationship between DFS and inflammatory markers.

**Figure 3.**
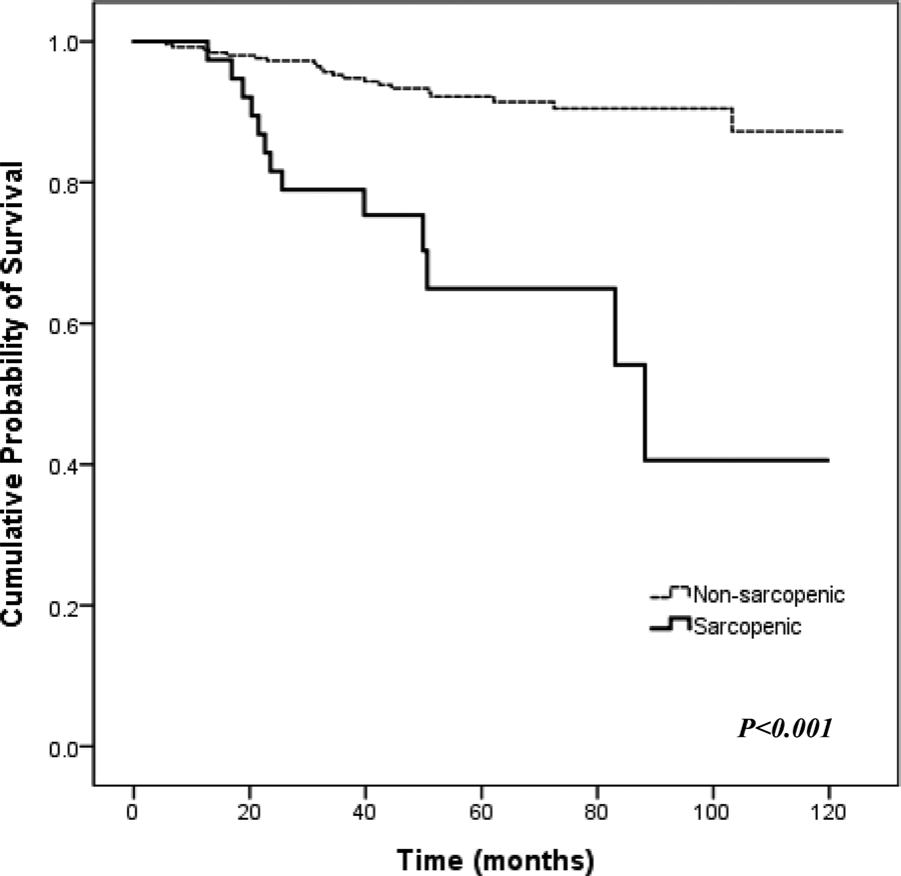
Disease-free survival (DFS) stratified by baseline sarcopenia among the enrolled patients (n = 293). The mean DFS of sarcopenic patients was lower than that of nonsarcopenic patients (89.8 months and 118.8 months, respectively, *P* < .001) according to the pectoral muscle area (PMA).

**Figure 4.**
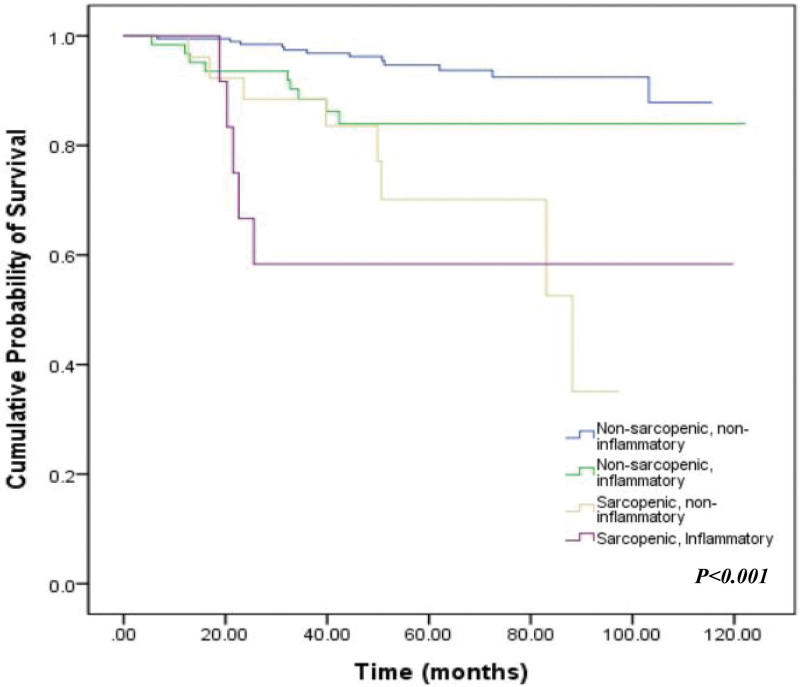
Disease-free survival (DFS) stratified by baseline sarcopenia and lymphocyte-to-monocyte ratio (LMR). Nonsarcopenic and noninflammatory patients were the most favorable group regarding DFS, whereas sarcopenic and inflammatory patients had worse outcomes.

As shown in Fig. 3, Sarcopenia based on PMA significantly shortened DFS in the enrolled patients (89.8 vs 118.8 months, *P* < .001). The mean DFS of inflammatory and noninflammatory patients based on LMR were 102.3 months (95% CI, 93–111.7 months) and 106.6 months (95% CI, 102.9–110.3 months), respectively (*P* = .011). Additionally, the cohort was divided into 4 groups according to sarcopenia and inflammatory status, and survival rates were compared between these groups (Fig. 4). Regardless of LMR status, patients in the PMA-sarcopenia group had a poorer prognosis than those in the nonsarcopenia group. Sarcopenic patients with a low LMR showed worse outcomes in DFS, while nonsarcopenic patients with a high LMR showed the best outcome in mean DFS (74.8 vs 109.6 months, respectively, *P* < .001).

### 3.3. Predictors of survival

Univariate comparisons revealed that the relapse of nonmetastatic breast cancer was significantly influenced by the positive lymph node stage at diagnosis, low LMR, and sarcopenia status (Table 2). In multivariate analysis, positive lymph node stage, low LMR, and sarcopenia status were independent poor prognostic factors for DFS. Univariate analysis showed that OS, N stage at diagnosis, low LMR, and sarcopenia defined by PMA were associated with a poor prognosis. N stage at diagnosis, low LMR, and sarcopenic status were confirmed as independent poor prognostic factors using multivariate analysis (Table 3). To clarify the correlation of LMR and PMA, we performed Chi-square test, and the result shown that there was no statistical correlation between the 2 factors (*P* = .336).

**Table 2:** Univariate and multivariate analysis for relapse of nonmetastatic breast cancer.

Characteristics	Univariate		Multivariate	
	HR (95% CI)	*P* value	HR (95% CI)	*P* value
Age (<50 vs ≥ 50 years)	0.47 (0.21–1.06)	.069	-	
T stage (T1 vs T2–4)	1.34 (0.62–2.9)	.460	-	
N stage (N0 vs N1–3)	**4.41** **(1.75**–**11.09**)	**.002**	**5.08** **(2.16**–**11.92**)	**<.001**
Operation method (MRM vs BCS)	0.98 (0.45–2.14)	.957		
Chemotherapy (no vs yes)	2.53 (0.2–6.25)	.83		
DM (no vs yes)	1.61 (0.67–3.89)	.291		
Obesity (no vs yes)	1.25 (0.59–2.62)	.56		
LMR (≥3.95 vs < 3.95)	**2.63** **(1.04**–**6.68**)	**.011**	**2.78 (1.32**–**5.91**)	**.007**
PMA (nonsarcopenic vs sarcopenic)	**3.63** **(1.07**–**12.35**)	**.038**	**3.80 (1.85**–**7.8**)	**<.001**

BCS = breast conserving surgery, CI = confidence interval, DM = diabetes mellitus, HR = hazard ratio, LMR = lymphocyte-to-monocyte ratio, MRM = modified radical mastectomy, PMA = pectoralis muscle area.

**Table 3:** Univariate and multivariate analysis for overall survival of nonmetastatic breast cancer patients.

Characteristics	Univariate		Multivariate	
	HR (95% CI)	*P* value	HR (95% CI)	*P* value
Age (<50 vs ≥ 50 years)	0.49 (0.15–1.64)	.251	-	
T stage (T1 vs T2–4)	2.03 (0.59–7.05)	.263	-	
N stage (N0 vs N1–3)	**12.98 (1.55**–**108.69**)	**.018**	**19.09** **(2.47**–**147.8**)	**.005**
Operation method (MRM vs BCS)	0.82 (0.28–2.39)	.711		
Chemotherapy (no vs yes)	2.18 (0.3–7.8)	.98		
DM (no vs yes)	1.58 (0.44–5.64)	.484		
Obesity (no vs yes)	1.07 (0.34–3.44)	.903		
LMR (≥3.95 vs < 3.95)	**2.38 (0.79**–**7.09**)	**.011**	**3.31** **(1.24**–**8.89**)	**.017**
PMA (nonsarcopenic vs sarcopenic)	**10.07 (0.95**–**106.22**)	**.05**	**6.28** **(2.36**–**16.72**)	**<.001**

BCS = breast-conserving surgery, CI = confidence interval, DMHR = hazard ratio, LMR = lymphocyte-to-monocyte ratio, MRM = modified radical mastectomy, PMA = pectoralis muscle area.

## 4. Discussion

In this study, we investigated the individual and combined prognostic effects of sarcopenia using PMA and systemic inflammatory status using LMR on DFS in women with nonmetastatic breast cancer. Multivariate analysis revealed sarcopenia and low LMR in predicting the prognosis of patients with shortened DFS. Although we found that combined sarcopenia and low LMR were significantly related to poor survival, sarcopenia had a greater influence on DFS than the inflammatory status based on low LMR.

To control the relapse risk in patients with breast cancer, it is crucial to predict the risk of disease relapse or patient death, and many efforts have been made to identify simple and low-cost prognostic factors. Therefore, many studies have investigated the usefulness of body composition measured by CT and peripheral blood parameters that reflect systemic inflammation as prognostic factors in women with breast cancer.^[19,20]^

To identify easily measurable prognostic factors among patients with breast cancer, we measured the sum of the peri-PMA to assess sarcopenia in patients with nonmetastatic breast cancer. As the pectoralis muscle is easy to measure, its area can be standardized across cohorts and has been reported to be associated with sarcopenia in patients with lung cancer.^[21,22]^ Our study suggests that this measure may be a good surrogate marker for sarcopenia in patients with nonmetastatic breast cancer.

Another important finding of the present study was that patients with a low LMR had worse outcomes in both the univariate and multivariate analyses. Moreover, patients with both low PMA and low LMR had significantly lower DFS than other patients, indicating that a combination of these factors can provide a stronger prediction of survival outcomes. The relationship between inflammatory parameters and prognosis of patients with cancer has been reported in previous studies. These parameters are easy to measure by collecting peripheral blood samples and can reliably reflect individualized systemic inflammatory status.^[23]^ A low LMR, which indicates systemic inflammatory status and worse survival outcomes, has been reported.^[24–26]^ Lymphocytes can infiltrate the tumor microenvironment and express various factors such as tumor-infiltrating lymphocytes, thereby affecting tumor immune responses.^[27–29]^ Monocytes can differentiate into tumor-associated macrophages, which have immunosuppressive effects and promote angiogenesis, tumor spread, and metastasis.^[30]^ Therefore, the LMR level can accurately identify the tumor immune response and can be considered a potential prognostic marker.

A major limitation of this study was its retrospective design, which made it difficult to generalize the results. Despite its retrospective design, the differences in DFS with or without sarcopenia and a low LMR were striking. Second, muscle strength measurements were not possible because of the study design. Additionally, the mass of the pectoralis muscle was measured by a trained researcher, which may not accurately reflect the entire muscle volume and the cutoff value of PMA for sarcopenia, which is defined below the ROC curve. The cutoff value of PMA for sarcopenia should be validated in future studies.

## 5. Conclusions

In conclusion, this is the first study to show that baseline sarcopenia assessed using PMA and low LMR is associated with poor prognosis in patients with nonmetastatic breast cancer who received adjuvant radiotherapy. This finding underscores the importance of studying the prognostic role of sarcopenia, especially in combination with parameters reflecting systemic inflammation, in patients with nonmetastatic breast cancer. These factors are expected to provide a new method for predicting the risk of relapse in patients with nonmetastatic breast cancer. Further prospective studies are required to confirm our results.

## Authors contributions

**Conceptualization:** Haa-Na Song, Bae-Kwon Jeong.

**Data curation:** Haa-Na Song, In Bong Ha.

**Formal analysis:** Ki Min Kang.

**Methodology:** Hoon Sik Choi.

**Validation:** Ju Yeon Kim, Jae Myung Kim, Jin Hee Jeong.

**Writing—original draft:** Haa-Na Song.

**Writing—review and editing:** Bae-Kwon Jeong.

## Supplementary Material


