# Supplementary figures and images for: Sarcopenia using pectoralis muscle area and lymphocyte-to-monocyte ratio (LMR) are independent prognostic factors in patients for nonmetastatic breast cancer
Source: Medicine (Baltimore). 2022 Dec 9;101(49):e32229. doi: 10.1097/MD.0000000000032229 (PMC9750599; doi:10.1097/MD.0000000000032229)

## Slide 1
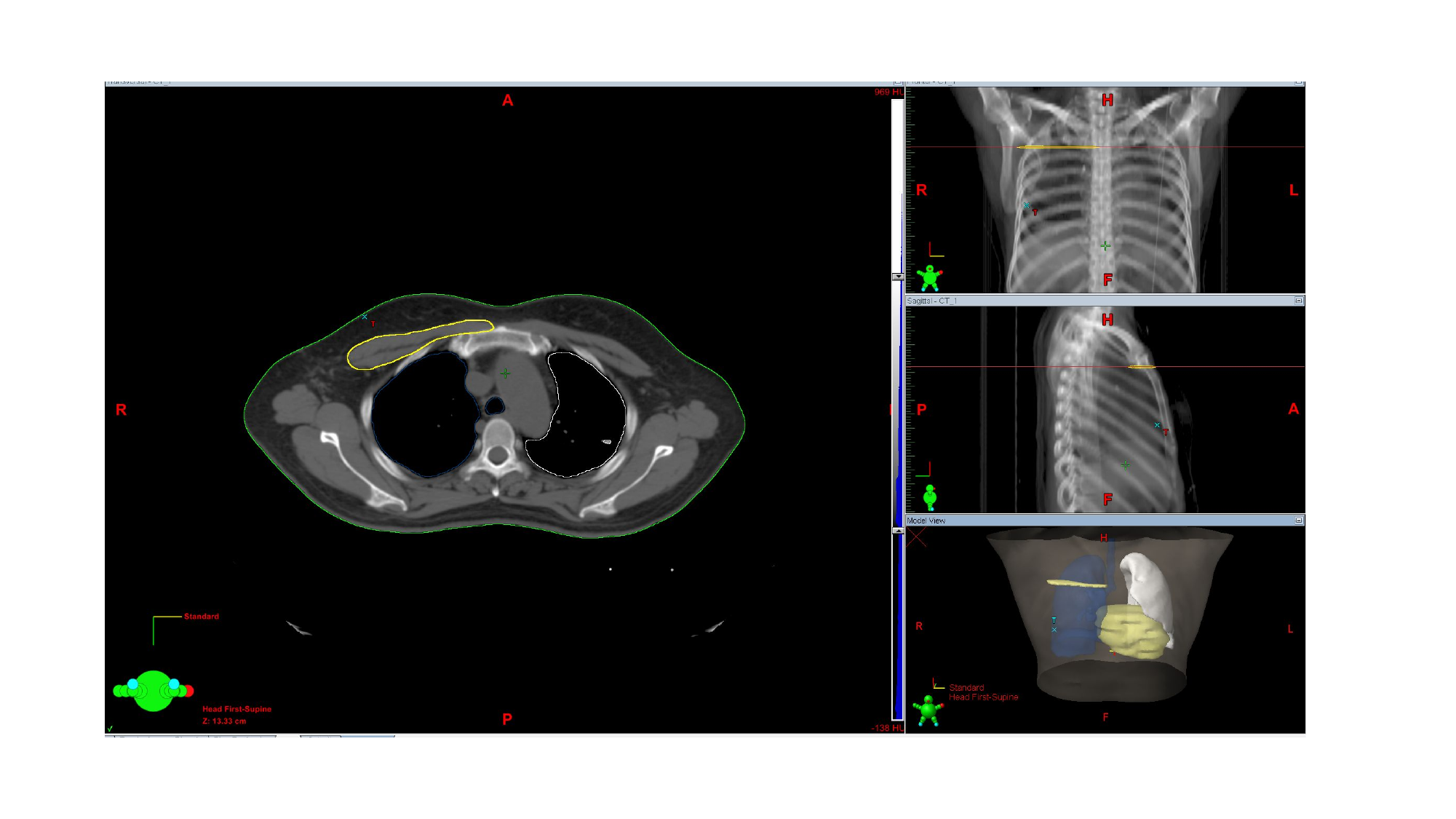

Supplement: Supplementary file 1 [file medi-101-e32229-s001.pptx]
